# Effect of 8-week of dietary micronutrient supplementation on gene expression in elite handball athletes

**DOI:** 10.1371/journal.pone.0232237

**Published:** 2020-05-01

**Authors:** Jorge Molina-López, María Antonieta Quispe Ricalde, Basilio Valladares Hernández, Antonio Planells, Roberto Otero, Elena Planells

**Affiliations:** 1 Department of Physiology, Faculty of Pharmacy, University of Granada, Granada, Spain; 2 Institute of Nutrition and Food Technology, Biomedical Research Center, Health Sciences Technological Park, University of Granada, Granada, Spain; 3 University Institute of Tropical Diseases and Public Health of the Canary Islands, University of La Laguna, La Laguna, Tenerife, Canary Islands, Spain; 4 Unit of Social Studies of the Defense, General Technical Secretary, Ministry of Defence, Madrid, Spain; 5 Department of Statistics. Faculty of Social and Legal Sciences, University of Carlos III, Getafe, Madrid, Spain; Universita degli Studi di Roma 'Foro Italico', ITALY

## Abstract

**Purpose:**

A study was made of the changes in gene expression in elite handball athletes, comparing gene modulation before, after and in the absence of an 8-week nutritional intervention with multivitamin/mineral supplements.

**Methods:**

Thirteen elite handball athletes (aged 22.9 ± 2.7 years) and 13 sedentary controls (aged 20.9 ± 2.8 years) were included. Three timepoints were established: T0 (baseline conditions); T8 (after 8 weeks of supplementation with a multivitamin/mineral complex); and T16 (after 8 weeks in the absence of supplementation). The expressions of a total 112 of genes were evaluated by RT-qPCR analysis with the QuantStudioTM 12K Flex Real-Time PCR System.

**Results:**

The analysis revealed different gene regulation profiles of genes implicated in cell communication, cell energy metabolism, inflammation and the immune system, oxidative stress and muscle function in athletes compared to sedentary controls under resting conditions (upregulated genes: effect size = large, ƞ2 = 1.011 to 1.398, p < 0.05; downregulated genes: effect size = large, ƞ2 = 0.846 and 1.070, p < 0.05, respectively). The nutritional intervention encouraged gene upregulation in elite athletes (p < 0.05). In a follow-up investigation, the IRAK1, CD81, ITGB1, ACADS PDHA2 and GPX1 genes were downregulated in athletes, with a moderate main effect for time-by-group interaction (ηP2 = 0.099 to 0.133; p < 0.05). Additionally, nutritional genes such as MTHFR and THTPA revealed a moderate effect over all the timepoints and group interaction in the study (ηP2 = 0.070 to 0.092; p < 0.05).

**Conclusions:**

Elite handball athletes showed a different expression profile in reference to key genes implicated in several sports performance-related functions compared to the sedentary controls, in addition to modulation of gene expression after multivitamin/mineral supplementation.

## Introduction

Nutrition has the potential to contribute to successful performance in elite athletes, and in this regard dietary supplements require special consideration in their nutritional strategies [1]. Exercise represents an important challenge for global body homeostasis, with acute and adaptive responses occurring at cellular and systemic level [2]. In this context, micronutrients play an important role in regulating processes ranging from energy production to the generation of new cells and proteins. The availability of nutrients contributes to marked differences in multiple functions such as the promotion of tissue repair, immune system support [3,4], and the reduction of oxidative stress and inflammation [5], and may enhance athletic performance in certain cases [6]. One important way to improve our understanding of these effects is by investigating the cellular and molecular responses to exercise.

The current challenge is to establish a paradigm through which deeper and more sophisticated comprehension of the complex physiological and metabolic networks of a multisystem organism can be gained [7]. Peripheral blood can potentially be used to explore the subtle changes that occur in individual body systems, in addition to contributing to the diagnosis of a variety of physiopathological conditions [8]. It is known that peripheral blood expresses numerous genes, and that the levels of expression are modified by genetic and environmental factors [9]. As a matter of fact, over 80% of the genes expressed in peripheral blood are also expressed by different human tissue types [10].

In the last decade there has been growing interest in understanding how the body responds to physical training at cellular, subcellular and molecular level [11–14], and in this context nutritional support could represent a primary factor affecting human performance [15]. Based on previous studies, a number of genes expressed in peripheral blood and involved in the response to endurance exercise have been identified in well trained athletes [12,13]. Using a microarray technique, Zieker et al. [12] identified a total of 36 genes (implicated in signal transduction, membrane protein expression, cell interaction/apoptosis, oxidative stress, immune system function and inflammation pathways) that experienced changes in expression immediately after exercise. Moreover, several studies [11,16] have used peripheral blood to clarify the effects of exercise on gene expression in healthy untrained men, as well as in elite athletes. Liu et al. [11] indicated that intense endurance exercise training can chronically induce transcriptional changes in peripheral blood leukocytes, upregulating genes related to protein production and mitochondrial energetics (OXPHOS), and downregulating genes involved in inflammatory responses among young healthy individuals.

The efficacy of supplementing the diet of athletes in order to ameliorate physical adaptations to training has been widely questioned [6]. Some studies [17–20] have shown that gene expression during exercise could be modulated by nutritional intake. For example, *in vivo* studies have observed that protein ingestion enhances post-exercise AAT expression [17], and that high protein diet supplementation causes increased AQP7 and GLUT4 expression in animal models [18]. In contrast, dietary almond and olive oil-based docosahexaenoic acid and vitamin E-enriched beverage supplementation resulted in no differences in antioxidant gene expression, athletic performance or oxidative stress markers [21]. *In vitro* studies, however, have suggested that the increased expression of antioxidant genes by peripheral blood mononuclear cells seems to be due to the response to postprandial oxidative stress generated after the consumption of a high fat diet [19]. To the best of our knowledge, no studies have analyzed the potential effects of nutritional supplementation upon gene expression in power sports such as handball. Therefore, the challenge is to determine how dietary supplementation may influence the metabolic response of proteins implicated in nutrient transport and the modulation of genes to promote physiological changes resulting from exercise. Additionally, there are still shortcomings in our understanding of the variations in gene expression observed in well-trained athletes compared to their untrained counterparts.

The present study involves the use of high-throughput real-time quantitative polymerase chain reaction (RT-qPCR) analysis, improving RNA quantification and internal standard selection, with two main objectives in mind: (a) to determine the modulation of gene expression in athletes with respect to sedentary controls under baseline conditions; and (b) to compare gene expression modulation after multivitamin/mineral supplementation. We hypothesized that there would be a different expression profile for genes implicated in exercise performance, and that a difference would be observed in the regulation of genes related to nutrient transport and metabolism between athletes and sedentary controls before and after the nutritional intervention.

## Materials and methods

### Participants

Thirteen elite handball athletes aged 22.9 ± 2.7 years (HAND group) and 13 sedentary controls aged 20.9 ± 2.8 years (SED group) were recruited in the present study. Although 32 participants were initially recruited, only 26 completed the study (**S1 Fig**). The 13 athletes belonged to the *Club Deportivo Balonmano Puente Genil*, which participated in Honor B Division of the Spanish Professional Handball League. They were all experienced athletes and had been training for 8 to 12 years.

The study was conducted during the competitive second phase of the season. Within the annual training period, the handball athletes had already developed general and specific preparation prior to competition. The latter began with a first period in which the athletes played a total of 12 games. The second competitive phase began two weeks after completion of the first phase, and during the two-week interval between phases the training duration of the athletes consisted of 10.1 ± 2.5 h/week of a handball training regimen including both indoor exercise and integrated conditioning exercises, in addition to competition in matches on weekends. The pre-designed weekly training schedule of the handball team comprised the following: Monday (strength training and individual technique); Tuesday (rest or strength); Wednesday (integrated strength training and small side games); Thursday (offensive and defensive technical-tactical training); Friday (activation training with technical-tactical training for matches); Saturday (match); Sunday (rest) (**Table 1**). The researchers did not interfere in the training program of the athletes. With regard to the 13 sedentary controls, physical activity was measured using the previously validated International Physical Activity Questionnaire (IPAQ) [22]. None reported at least 150 min/week of moderate intensity or 75 min/week of vigorous intensity aerobic physical activity, necessary for procuring substantial health benefits [23].

**Table 1:** Contents of weekly training sessions in handball athletes.

**Monday**	• Scouting session. • Lower and upper strength training: Multi-joint barbell or resisted exercises such as squat, lunges, deadlift, hip thrust, bench press, overhead press exercises 3–5 × 4–6 reps loaded 30–70% 1 RM Rec: > 2′ Total duration: 45 min • Team handball training with emphasis on individual technique in reduced situations: 1x1, 2x1 and 2x2 change in direction, throwing drills, acceleration and passing exercises. At the end of the session, ball circulation exercises. Total duration: 75 min
Strength training and individual technique	
**Tuesday**	• Multi-joint strength training: Olympic weightlifting such as cleans, power cleans, snatches, jerks 3–5 × 1–2 reps loaded >85% 1 RM Rec: > 3′ Total duration: 60 min
Rest or strength	
**Wednesday**	• Concurrent strength training: Bodyweight hops, jumps drop jumps, medicine ball throws, bench throws, push presses 3–5 × 6–10 reps loaded 30–50% 1 RM Rec: > 2′ • Team handball training: small side games 3x3, 4x4 and situations of numerical superiority and inferiority and counterattack and dynamic withdrawal emphasis. At the end of the session, offense and defense against 6:0, 5:1 or 3:2:1. Technical/tactical strategies. Total duration: 90 min
Integrated strength training and small side games	
**Thursday**	• Team handball training: offense and defense against 6:0, 5:1 or 3:2:1. Technical/tactical strategies in match competition. Total duration: 90 min
Offensive and defensive technical-tactical training	
**Friday**	• Team handball training technical/tactical pre-match. Change in direction, throwing drills, acceleration exercises. Power play 6:0 or 5:1 defense with high intensity and low duration. Offense against 6:0, 5:1 or 3:2:1 + fast break. Total duration: 75 min.
Activation training with technical-tactical training for matches	
**Saturday**	• Match
Match	
**Sunday**	• Rest
Rest	

The inclusion criteria were: passing of a recruitment medical evaluation consisting of a clinical examination, evaluation of the medical history, non-smoker status and the absence of medications use. The exclusion criteria were: consumption of nutritional supplements of any kind in the 6 weeks before and during the study, and no desire to participate in the study. The study was approved by the Ethics Committee of the University of Granada, and was conducted in accordance with the Declaration of Helsinki. The purpose of the study and its possible risks and discomforts were explained to the participants before their written consent was obtained. The study was registered at the US National Institutes of Health (ClinicalTrials.gov) NCT03672786. Title: Gene expression in intervened athletes.

### Experimental design

**Fig 1:**
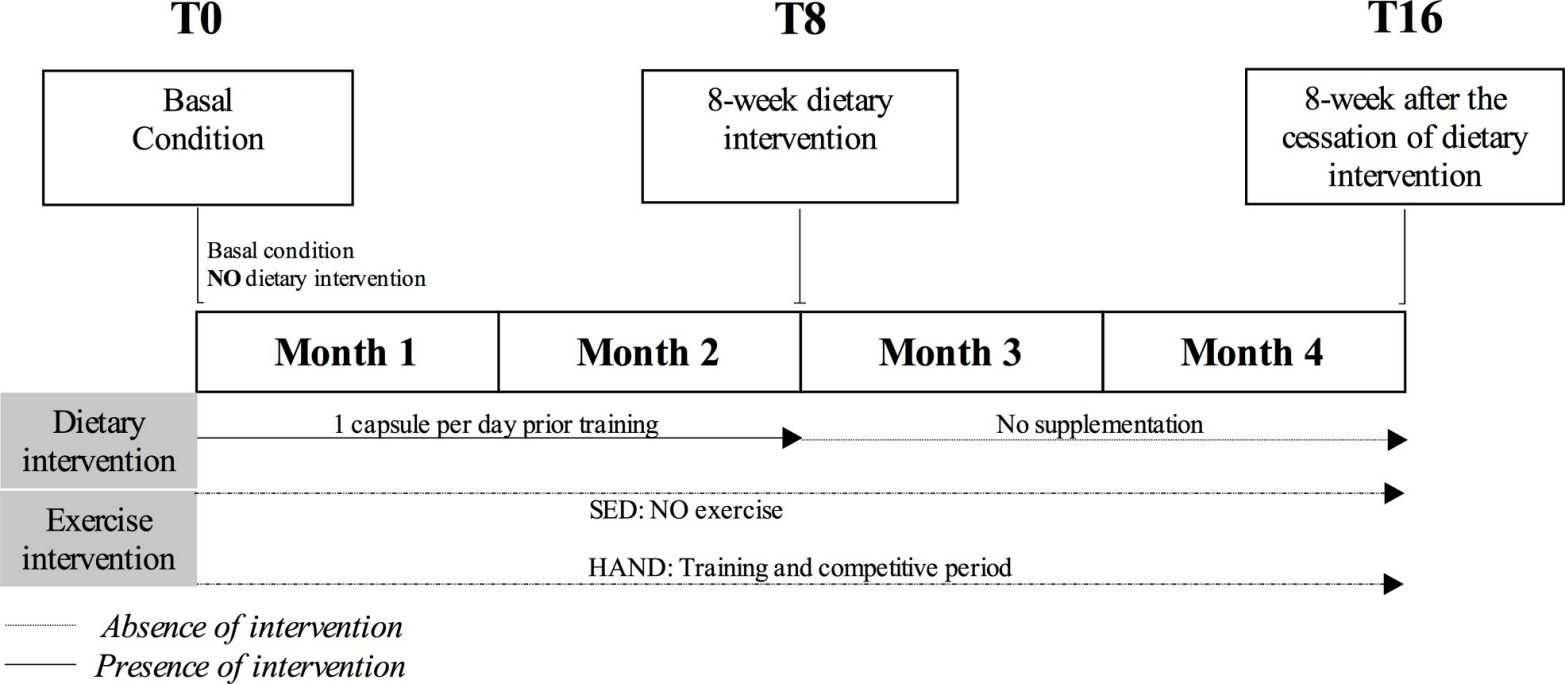
Study design. SED = Sedentary controls; HAND = Elite handball players.

A total of 26 healthy males agreed to participate in the present intervention study, which was carried out over a period of 8 weeks, with the definition of three evaluation timepoints: T0 (baseline conditions in the sedentary and handball groups); T8 (after 8 weeks of multivitamin/mineral supplementation); and T16 (after 8 weeks in the absence of the nutritional intervention) (**Fig 1**). The athletes were requested to maintain their normal training program during the study period. Daily intake was recorded during the study, and no significant differences in macro- and micronutrient intake were observed between the groups. The dietary intervention consisted of the oral administration of one daily multivitamin/mineral complex capsule (Multicentrum® Pfizer, Barcelona, Spain) before exercise during the controlled dietary intervention period. Multivitamin/mineral complex intervention adherence/compliance was defined as the percentage of all of the supplement capsules ingested throughout the study period. The participants completed a log to verify compliance (handball athletes = 90.4 ± 4.91%, sedentary controls = 91.5 ± 4.38%); two individuals in the sedentary control group failed to adhere to the supplementation protocol and were thus excluded from all analyses. On the other hand, biochemical and clinical-nutritional parameters were recorded at the beginning and at the end of the study to evaluate the safety of the product and assess adverse effects. The proportion of each vitamin and mineral in the supplement and the respective estimated adult Recommended Daily Allowances (% RDAs) are detailed in **S1 Table**.

### Participant characteristics

Body composition measurements were obtained by multi-frequency bioelectrical impedance (Tanita MC-980MA Multi-frequency Segmental Body Composition Analyzer, Barcelona, Spain). The participants were informed in advance of the conditions that had to be observed: no alcohol consumption for at least 24 hours before measurement, no vigorous exercise for at least 12 hours before measurement, no food or drink for at least three hours before measurement, and no urination immediately before measurement. All measurements took place in the morning at the same time. The following measurements were obtained: weight, body mass index (BMI) and fat mass expressed as the percentage of total fat in the body. Nutritional assessment of energy and micronutrient intake was quantitatively performed by means of a 72-hour dietary recall questionnaire. Recall accuracy was recorded with a set of photographs of prepared foods and dishes that are commonly consumed in Spain. The validated Nutriber® software package was used to estimate the intake of each nutrient for the individual athletes and sedentary controls [24]. Micronutrient intake was expressed as nutrient-density per 1000 kcal of energy. All subjects were required to maintain their nutritional habits during the study period. The physical characteristics of the participants are described in **Table 2**.

**Table 2:** Sample characteristics.

Measurement	Athletes	Sedentary
Age (years)	22.9 ± 2.7	20.9 ± 2.8
Height (m)	1.87 ± 0.06	1.78 ± 5.91
Weight (kg)	86.7 ± 5.36	78.2 ± 7.49
Body mass index (kg·m2^-1^)	24.7 ± 1.12	24.7 ± 2.83
Body fat (%)	11.6 ± 2.53	17.1 ± 4.64
Compliance (%)	90.4 ± 4.91	91.5 ± 4.38
*Dietary intake*		
Energy (kcal·day^-1^)	2974.5 ± 211.1*	2177.4 ± 488.1
Vitamin A (μg·day^-1^)	300.9 ± 96.8	360.8 ± 141.9
Vitamin E (mg·day^-1^)	3.50 ± 1.30	4.81 ± 0.82
Vitamin C (mg·day^-1^)	39.9 ± 17.6	45.1 ± 23.1
Vitamin B1 (mg·day^-1^)	0.84 ± 0.17	0.78 ± 0.32
Vitamin B2 (mg·day^-1^)	0.90 ± 0.21	0.94 ± 0.36
Vitamin B6 (mg·day^-1^)	0.97 ± 0.23	0.99 ± 0.32
Vitamin B12 (μg)	2.50 ± 0.58	3.41 ± 2.21
Vitamin D (μg)	1.78 ± 0.88	2.08 ± 0.59
Biotin (μg·day^-1^)	2.32 ± 0.81	3.04 ± 2.16
Folate (μg·day^-1^)	101.5 ± 29.6	108.6 ± 37.1
Niacin (mg·day^-1^)	13.1 ± 2.63	11.3 ± 3.65
Pantothenic acid (mg·day^-1^)	2.51 ± 0.54	2.38 ± 0.59
Calcium (mg·day^-1^)	421.3 ± 112.7	442.9 ± 118.3
Phosphorous (mg·day^-1^)	566.0 ± 104.2	633.7 ± 78.6
Magnesium (mg·day^-1^)	126.2 ± 41.7	147.6 ± 39.9
Iron (mg·day^-1^)	7.56 ± 2.06	7.75 ± 1.84
Iodine (μg·day^-1^)	35.6 ± 24.1	35.4 ± 16.9
Cooper (μg·day^-1^)	0.52 ± 0.27	0.39 ± 0.15
Manganese (mg·day^-1^)	1.55 ± 0.81	2.05 ± 1.90
Selenium (μg·day^-1^)	32.3 ± 7.10	31.67 ± 2.90
Zinc (mg·day^-1^)	3.98 ± 3.29	6.27 ± 3.70
*Biochemical parameters*		
Glucose (mg·dL^-1^)	85.15 ± 5.67	83.12 ± 5.82
Creatinine (mg·dL^-1^)	0.92 ± 0.11	0.88 ± 0.07
Transferrin (mg·dL^-1^)	261.21 ± 27.81	274.93 ± 38.71
Prealbumin (mg·dL^-1^)	26.76 ± 3.53	29.01 ± 6.39
Albumin (mg·dL^-1^)	4.68 ± 0.15	5.01 ± 0.31
HDL (mg·dL^-1^)	58.28 ± 13.58	52.25 ± 7.46
LDL (mg·dL^-1^)	74.00 ± 22.89	93.25 ± 23.85
Triglycerides (mg·dL^-1^)	64.50 ± 26.59	75.06 ± 27.47
Total cholesterol (mg·dL^-1^)	147.86 ± 26.74	160.43 ± 25.81

All data are expressed as the mean and standard deviation (SD). Micronutrient intake was expressed as nutrient-density per 1000 kcal of energy.

* p < 0.05 Statistically significant differences.

### Blood sampling and RNA isolation and quantification

The participants reported to the laboratory on Monday morning between 8 a.m. and 10 a.m. after abstaining from physical exercise for at least 24 hours. After a supine resting period of 10 minutes, venous blood samples were collected from the antecubital vein using standard techniques. Specifically, samples were collected in special tubes (PAXgene blood RNA tube, Becton Dickinson, Germany) to determine RNA expression, and were stored at -80°C until analysis.

The PAXgene blood RNA tubes were incubated for two hours at room temperature in order to achieve complete lysis of blood cells. Then, 2.5 ml of human whole blood in PAXgene blood RNA tubes was centrifuged for 10 minutes at 5000 g. After centrifugation, the supernatant was discarded and the pellet was washed with 4 ml of RNase-free water and vortexed and centrifuged for 10 minutes at 5000 g. After washing, the pellet was dissolved in 350 μl resuspension buffer and incubated with 300 μl binding buffer and 40 μl proteinase K for 10 min at 55°C in a shaker-incubator. The lysate was transferred to a PAXgene shredder spin column and centrifuged at 18,000 g for three minutes. The flow-through fraction was mixed with 350 μl ethanol and transferred to a PAXgene RNA spin column. After washing the column with washing buffer 1, samples were incubated with 10 μl of DNase I for 15 minutes. The PAXgene RNA spin columns were washed with washing buffer and RNA eluted with 40 μl elution buffer. The effectiveness of the DNase treatment was assessed in RT-negative samples.

The RNA yield was estimated by measuring absorbance at 260 nm in a NanoDrop ND-1000 spectrophotometer (Thermo Fisher Scientific). RNA purity was calculated from the ratio of absorbance at 260 nm and 280 nm. The OD 260/280 ratio was between 1.9 and 2.2 for all samples. RNA integrity was assessed using the Eukaryote Total RNA Nano assay on an Agilent 2100 Bioanalyzer (Agilent Technologies). RNA quality was rated according to the RNA integrity number (RIN) using the 2100 Bioanalyzer (Agilent Technologies). The high quality samples (RIN ≥ 9) and standard quality samples (RIN = 7) were included in the study to secure a more realistic approach.

### High-throughput real-time quantitative PCR (RT-qPCR)

Reverse transcription was performed with random hexamers using the High Capacity cDNA Reverse Transcription Kit (Life Technologies, Carlsbad, CA, USA) according to the instructions of the manufacturer. Two μg of the total RNA was reverse transcribed with random hexamers using the High Capacity cDNA Reverse Transcription Kit. Thermal cycler conditions were set to run for two hours at 37°C and for 10 minutes at 75°C, followed by rapid cooling to 4°C.

Real-time PCR was performed using the QuantStudio 12K Flex Real-Time PCR System (Life Technologies, Carlsbad, CA, USA) with OpenArray panels using the QuantStudio 12K Flex Real-Time PCR System (Life Technologies, Carlsbad, CA, USA). One hundred and twelve TaqMan gene expression assays (**S2 Table**) (Applied Biosystems) included in the array were selected according to previous studies for their implication in nutrient transport [12,13,25–27] and different metabolic pathways related to sports performance (e.g., cardiovascular, inflammatory, mitochondrial, oxidative stress and other mechanisms) [2,28–31]. All TaqMan assays (primers and probe) were of commercial grade. The OpenArray panel features 3072 nano through-holes arranged into 48 subarrays per plate and 64 through-holes per subarray. Each subarray contains 56 amplicons that can be tested for one sample. A 1.2 μl volume of cDNA- or DNase-free water was mixed with 3.8 μl of TaqMan Gene Expression Master Mix (Applied Biosystems). Five μl of PCR mix was accurately added to each subarray using AccufillTM (Applied Biosystems). The OpenArray system conducts each PCR reaction in 33 nl of reagents. Each sample was analyzed in triplicate. Standard cycling conditions were used as recommended by the manufacturer. Thermal cycling and fluorescence detection were performed using the QuantStudio 12K Flex Real-Time PCR System (Applied Biosystems).

A previous study was carried out to select the most appropriate housekeeping genes (**S3 Table**). A total of 52 genes were included in the analysis in order to determine the stability value (M) and the coefficient of variation of the normalized reference gene expression levels using qBasePLUS software (Biogazelle, Gent, Belgium). The Cq value of every sample was loaded in qBasePLUS software in triplicate, with set cut-off values of M < 0.5 and V < 0.15, following the same methodology as previously published [32]. The selected housekeeping genes were: ATP5B (ATP synthase H+ transporting, mitochondrial F1 complex, beta polypeptide; NC_000076.6; gene expression assay Hs00969569_m1, Applied Biosystems); LUC7L2 (LUC7 like 2, pre-RNA splicing factor; NC_000007.14; gene expression assay Hs00255388_m1, Applied Biosystems); ARF1 (ADP-ribosylation factor 1; NC_000001.11; gene expression assay Hs00734523_m1, Applied Biosystems); and PAPOLA (Poly(A) polymerase alpha; NC_000014.9; gene expression assay Hs00413685_m1, Applied Biosystems). Crossing of threshold (Ct) values obtained for the target gene were normalized against ATP5B, LUC7L2, ARF1 and PAPOLA Ct values.

### Data analysis

The threshold cycle (Ct) values from the 26 samples analyzed were exported using the Expression Suite for QuantStudio 12K Flex Real-Time PCR System (Applied Biosystems, Thermo Fisher Scientific, Carlsbad, CA, USA). The software calculates Cq values using an algorithm that takes into account the efficiency of each individual curve, called the relative threshold cycle (Crt) method [33]. The Crt method sets an individual threshold for each curve that is based on the shape of the amplification curve, regardless of the height or variability of the curve in its early baseline fluorescence. The method first estimates a curve that models the reaction efficiency from the amplification curve. It then uses this curve to determine the Crt from the amplification curve, eliminating the need for “conventional” real-time PCR to calculate the efficiency of each reaction. Therefore, Cq values produced by this platform are already corrected for the efficiency of the amplification. Relative quantification was determined using the 2–ΔCT method [34]. Standardizing of the variables was carried out using the Shapiro-Wilk test, and homoscedasticity was assessed with the Levene test. The baseline dCt values were subjected to an unpaired t-test for baseline athlete and sedentary control comparisons. Effect sizes between the sedentary and athlete groups were calculated using Cohen’s d, and effect sizes were interpreted as small (d between 0.2–0.5), moderate (d between 0.5–0.8) or large (d > 0.8)—with effect sizes below 0.2 being considered trivial [35]. Two-way repeated-measures analysis of variance (ANOVA) was used to determine the effect of time (treatment or no treatment) and the interaction between time*group. The effect size was estimated using partial eta squared (η2, the proportion of variance in the outcome explained). Following Cohen, we interpreted the estimated η2 values as follows: small = 0.01; moderate = 0.06; large = 0.14 [35].

## Results

### Participants

Thirty-two individuals were initially eligible for inclusion in the study. Of these, two corresponding to the sedentary controls group were excluded because they reported to be physically active (**S1 Fig**). Of the 14 athletes allocated to intervention, one was excluded during follow-up due to injury. In turn, two of the sedentary subjects were excluded because they did not follow the multivitamin/mineral intervention, and one participant was excluded due to time commitment problems. Thus, 13 elite handball athletes and 13 sedentary controls were finally enrolled in the study.

On average, the elite handball athletes ingested significantly more energy than the sedentary controls (2974.5 ± 211.1 versus 2177.4 ± 488.1 Kcal; p < 0.05). Nutrient-density adjustment revealed no significant differences between the groups in any of the studied micronutrients. Biochemical profiles were within the reference values for healthy humans in both the elite athletes and in the sedentary controls.

### Gene expression analysis

To understand the influence of nutritional supplementation with multivitamin/mineral complex upon athletes compared to sedentary controls, a total of 112 genes related to both sports performance and nutrient metabolism were analyzed in whole blood at timepoints T0, T8 and T16. **S2 Table** shows a predesigned qPCR reaction of 112 genes related to sports performance and nutrient metabolism that are constitutively expressed by most cells, since they are genes that encode for proteins needed for cell function. All blood samples were analyzed according to whether they were obtained from age- and gender-matched sedentary controls or handball athletes and whether they were drawn at timepoints T0, T8 or T16. Of the 112 tested genes, 34 were not expressed in any of the analyzed blood samples. Overall, a total of 78 genes were expressed in all 26 analyzed blood samples. For further analyses, we extracted all genes that showed an absolute expression fold change of at least 1.5 in any of the abovementioned comparisons.

### Identification of gene expression response in athletes under resting conditions

The two groups were initially compared to assess the expression profile of trained athletes with respect to sedentary controls. During this period, the athletes were actively participating in the second competition period of the annual season, while the sedentary controls did not perform any type of physical activity. **Table 3** describes the regulatory profiles of RNA with fold change > 1.5 under baseline conditions in the athletes. A separate comparative t-test for independent samples considering the athletes and sedentary controls showed ITGB1, IL6R, MAPKAPK2, ACVR1B and SOD2 to be upregulated in athletes under resting conditions (effect size = large; ƞ2 = 1.011 to 1.398; p < 0.05). In contrast, PDHA2 and MAPK11 (P38) genes were downregulated in athletes under resting conditions (effect size = large; ƞ2 = 0.846 and 1.070; p < 0.05, respectively) versus the sedentary controls. Although the IFNG and MT3 genes showed a fold change > 1.5, this was not statistically significant between the groups.

**Table 3:** Gene expression in handball athletes compared to sedentary controls under resting conditions.

Gene id	Gene name	Function	Fold	Sd	Cohen’s d	*P Value*
IFNG	Interferon gamma	Acute inflammation	-1.53	0.26	0.529	*0*.*196*
IFNGR1	Interferon gamma receptor 1		-1.06	0.55	0.156	*0*.*695*
IRAK1	Interleukin-1 receptor-associated kinase 1		1.34	0.39	0.474	*0*.*269*
TANK	TRAF family member-associated NFKB activator		-1.04	0.59	0.114	*0*.*775*
CD81	CD81 molecule	Cell communication	1.33	0.47	0.572	*0*.*165*
ITGAX	Integrin alpha X (complement component 3 receptor 4 subunit)		1.27	0.60	0.757	*0*.*076*
**ITGB1**	**Integrin beta 1**		**1.69**	**0.51**	**1.230**	***0*.*005***
ACADS	Acyl-CoA dehydrogenase, C-2 to C-3 short chain	Cell energy metabolism	-1.17	0.43	0.301	*0*.*451*
**PDHA2**	**Pyruvate dehydrogenase (lipoamide) alpha 2**		**-5.62**	**0.04**	**0.846**	***0*.*042***
ACSL1	Acyl-CoA synthetase long-chain family member 1	Cholesterol homeostasis/metabolism	1.24	0.61	0.620	*0*.*134*
CD36/FAT	CD36 molecule (thrombospondin receptor)		-1.04	0.62	0.121	*0*.*762*
PPARGC1B	Peroxisome proliferator-activated receptor gamma, coactivator 1 beta		1.38	0.48	0.755	*0*.*070*
SCARB1	Scavenger receptor class B, member 1		-1.03	0.62	0.099	*0*.*803*
ALDOC	Aldolase C, fructose-bisphosphate	Glucose homeostasis/metabolism	1.00	0.61	0.005	*0*.*991*
KLF4	Kruppel-like factor 4 (gut)		1.01	0.35	0.017	*0*.*968*
PDK4	Pyruvate dehydrogenase kinase, isozyme 4		1.29	0.49	0.587	*0*.*150*
PRKAA1	Protein kinase, AMP-activated, alpha 1 catalytic subunit		1.05	0.77	0.261	*0*.*516*
CD130	Il-6 signal transducer	Immune/Inflammation	1.25	0.49	0.485	*0*.*240*
IGJ	Immunoglobulin J polypeptide, linker protein for immunoglobulin alpha and mu polypeptides		-1.03	0.31	0.040	*0*.*919*
IL1B	Interleukin 1		1.08	0.48	0.159	*0*.*691*
IL1RN	Interleukin 1 receptor antagonist		-1.16	0.68	0.561	*0*.*173*
**IL6R**	**IL-6 receptor**		**1.62**	**0.56**	**1.398**	***0*.*002***
JACK1	Janus kinase 1		1.40	0.59	0.948	*0*.*031*
JACK2	Janus kinase 2		1.04	0.58	0.102	*0*.*797*
**MAPK11 (P38)**	**Mitogen-activated protein kinase-activated protein kinase 11**		**-2.19**	**0.29**	**1.070**	***0*.*031***
**MAPKAP K2**	**Mitogen-activated protein kinase-activated protein kinase 2**		**1.62**	**0.48**	**1.048**	***0*.*032***
RPS6KB1	Ribosomal protein S6 kinase		-1.02	0.75	0.070	*0*.*863*
SELL	Selectin L		-1.16	0.78	0.832	*0*.*054*
SOCS3	Suppressor of cytokine signaling 3		1.31	0.35	0.422	*0*.*333*
STAT1	Signal transducer and activator of transcription 1, 91 kDa		1.06	0.61	0.159	*0*.*693*
STAT3	Signal transducer and activator of transcription 3		1.46	0.61	1.205	*0*.*007*
CDC20	Cell division cycle 20 homolog	Mineral homeostasis/metabolism	1.06	0.35	0.091	*0*.*821*
HFE	Hemochromatosis		1.07	0.56	0.149	*0*.*725*
MZB1	Marginal zone B and B1 cell-specific protein		1.17	0.38	0.198	*0*.*619*
SBP2	SECIS binding protein 2		-1.02	0.72	0.081	*0*.*840*
SLC30A1	Solute carrier family 30 (zinc transporter), member 1		1.13	0.77	0.666	*0*.*112*
TRPM6	Transient receptor potential cation channel, subfamily M, member 6		1.14	0.55	0.301	*0*.*457*
TRPM7	Transient receptor potential cation channel, subfamily M, member 7		-1.05	0.54	0.131	*0*.*743*
TXNDC5	Thioredoxin domain containing 5 (endoplasmic reticulum)		1.21	0.54	0.471	*0*.*260*
**ACVR1B**	**Activin A receptor, type IB**	Muscle growth/size/body function	**1.90**	**0.34**	**1.011**	***0*.*017***
GYS 1	Glycogen synthase 1 (muscle)		-1.01	0.38	0.009	*0*.*984*
UCP3	Uncoupling protein 3 (mitochondrial, proton carrier)		1.04	0.49	0.090	*0*.*821*
HIF1A	Hypoxia inducible factor 1, alpha subunit	Nuclear	1.37	0.65	1.134	*0*.*008*
PPARA	Peroxisome proliferator-activated receptor alpha		1.17	0.63	0.491	*0*.*267*
CAT	Catalase	Oxidative stress	1.09	0.73	0.406	*0*.*314*
GPX1	Glutathione peroxidase I		1.18	0.46	0.330	*0*.*443*
GPX4	Glutathione peroxidase 4		1.04	0.63	0.132	*0*.*741*
GSR	Glutathione reductase		1.33	0.73	1.390	*0*.*005*
HSP70	Heat Shock protein 70kDa protein 8		-1.24	0.65	0.745	*0*.*081*
HSPB1/HSP27	Heat shock 27kDa protein 1		-1.14	0.47	0.292	*0*.*525*
MT1F	Metallothionein 1F		-1.16	0.20	0.139	*0*.*742*
MT1G	Metallothionein 1G		1.01	0.12	0.009	*0*.*982*
MT2A	Metallothionein 2A		-1.08	0.25	0.087	*0*.*835*
MT3	Metallothionein 3		-1.77	0.05	0.321	*0*.*428*
PRDX5	Peroxiredoxin 5		1.19	0.62	0.530	*0*.*208*
SOD1	Superoxide dismutase 1		-1.07	0.71	0.289	*0*.*473*
**SOD2**	**Superoxide dismutase 2**		**1.58**	**0.59**	**1.298**	***0*.*005***
AK1	Adenylate kinase 1	Vitamin metabolism/transport	1.12	0.52	0.275	*0*.*491*
CTH	Cystathionase (cystathionine gamma-lyase)		-1.08	0.49	0.163	*0*.*682*
DHFR	Dihydrofolate reductase		-1.34	0.26	0.372	*0*.*396*
FTH1	Ferritin, heavy polypeptide 1		1.03	0.50	0.073	*0*.*863*
GGH	Gamma-glutamyl hydrolase (conjugase, folylpolygammaglutamyl hydrolase)		-1.41	0.47	0.707	*0*.*088*
MTHFD1	Methylenetetrahydrofolate dehydrogenase (NADP+ dependent) 1		-1.01	0.65	0.043	*0*.*914*
MTHFD1L	Methylenetetrahydrofolate dehydrogenase (NADP+ dependent) 1-like		-1.23	0.55	0.550	*0*.*177*
MTHFR	Methylenetetrahydrofolate reductase (NADPH)		1.25	0.52	0.572	*0*.*166*
MTR	5-methyltetrahydrofolate-homocysteine methyltransferase		1.18	0.69	0.569	*0*.*163*
MTRR	5-methyltetrahydrofolate-homocysteine methyltransferase reductase		-1.16	0.64	0.487	*0*.*231*
SHMT1	Serine hydroxymethyltransferase 1 (soluble)		1.13	0.47	0.235	*0*.*556*
SLC11A2	Solute carrier family 19 (thiamine transporter), member 2		-1.01	0.71	0.023	*0*.*956*
SLC19A1	Solute carrier family 19 (folate transporter), member 1		1.43	0.37	0.637	*0*.*152*
SLC19A2	Solute carrier family 19 (thiamine transporter), member 2		-1.10	0.54	0.262	*0*.*511*
TCN1	Transcobalamin I (vitamin B-12 binding protein, R binder family)		-1.23	0.47	0.399	*0*.*320*
TCN2	Transcobalamin II		-1.01	0.56	0.003	*0*.*995*
THTPA	Thiamine triphosphatase		1.15	0.53	0.296	*0*.*459*
TPK1	Thiamin pyrophosphokinase 1		1.10	0.56	0.019	*0*.*961*
VDR	Vitamin D (1,25- dihydroxyvitamin D3) receptor		1.41	0.37	0.472	*0*.*248*

FC, fold change, was calculated as the ratio of RNA levels (athletes/sedentary). Data are for mean and standard deviation fold change. The cut-off calculation score to establish a significant fold change was established as 1.5. Cohen’s d effect size were also performed to determine the effect of athletic status (small: *d* between 0.2 and 0.5; moderate: *d* between 0.5 and 0.8; and large: *d* > 0.8), with effect sizes below 0.2 considered trivial [35]. Differences were considered significant at *p*-values < 0.05.

### Comparison of gene expression patterns after multivitamin/mineral intervention

**Fig 2:**
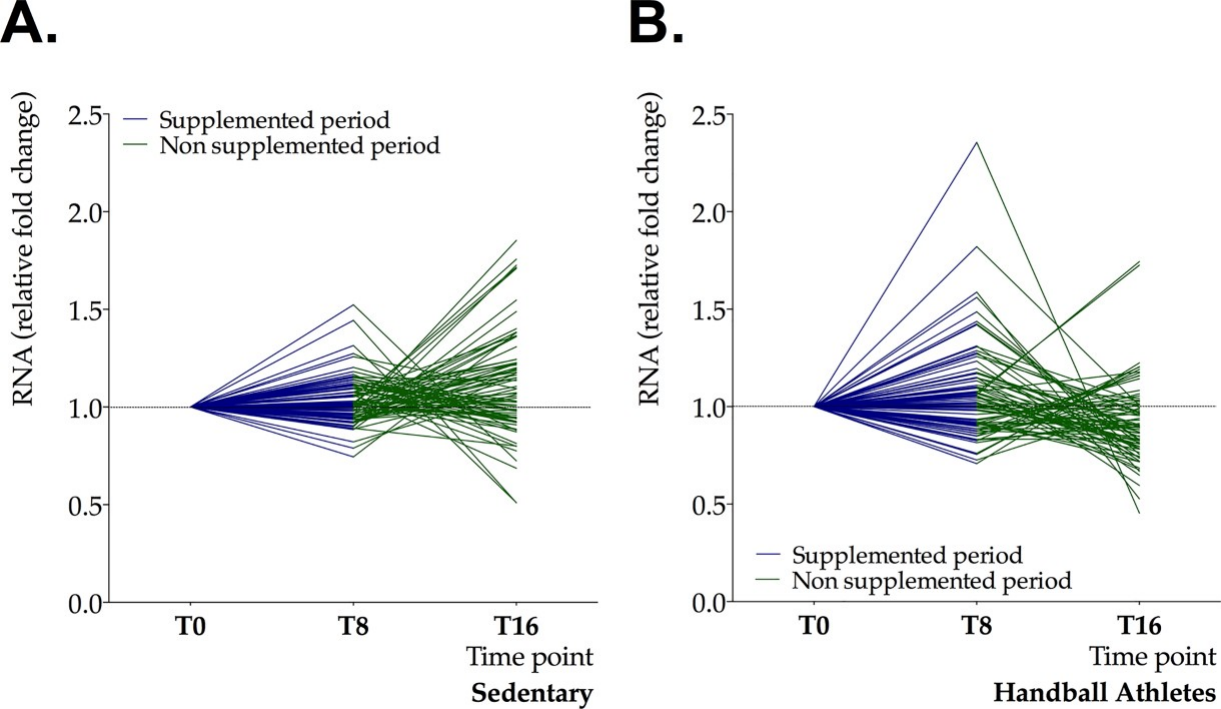
Relative fold changes in RNA levels of 78 genes in sedentary controls (A) and handball athletes (B) after 8 weeks of multivitamin/mineral intervention (blue lines—T8) and after 8 weeks in the absence of nutritional intervention (green lines—T16).

**Fig 2** shows the variation over time of the RNA levels corresponding to the 78 genes analyzed, as well as the genes showing significant differences during the study period. The athletes showed a tendency to upregulate some key genes at timepoint T8 in comparison to the sedentary controls. In contrast, the handball athletes showed a tendency towards down-expression at T16, while the controls exhibited greater variability in gene expression response at that same timepoint.

**Fig 3:**
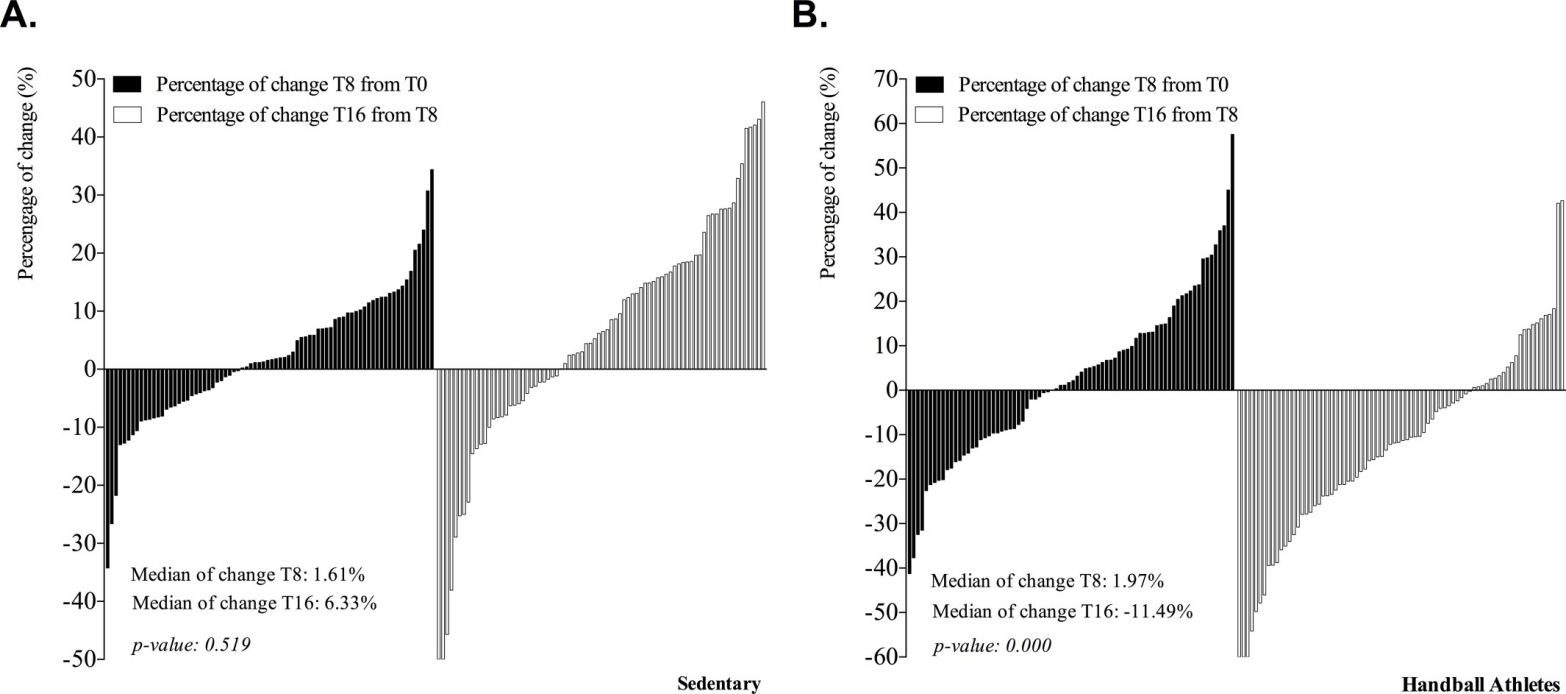
Percentage changes in gene expression in sedentary controls (A) and handball athletes (B) from baseline (T0) to after 8 weeks of nutritional intervention (T8) and after 8 weeks in the absence of nutritional intervention (T16).

The percentage changes in RNA levels corresponding to the 78 genes analyzed in the sedentary controls versus the baseline values were not significant at either timepoint T8 or timepoint T16 (median variation at T8 and T16: 1.61% and 6.33%, respectively) (**Fig 3**). In the case of the handball athletes, total gene response showed no significant changes at T8 (median variation at T8: 1.97%). However, the general analysis of gene expression from baseline to timepoint T16 (8 weeks in the absence of nutritional intervention) showed a significant decline (median variation at T16: -11.49%).

Repeated measures ANOVA was performed to compare the modulation in gene expression before and after multivitamin/mineral supplementation and analyze the interaction between time and groups. **Table 4** shows the RNA regulatory profiles at baseline (T0) versus nutritional intervention (T8) and after 8 weeks in the absence of nutritional intervention (T16), in both the sedentary controls and elite athletes. Main effect of time was revealed for key genes such as ITGB1, PDHA2 and ACVR1B, involved in sports performance functions such as cell energy metabolism and muscle growth/size/body function (ηp2 = 0.102, p < 0.006; ηp2 = 0.132, p < 0.002 and ηp2 = 0.113, p < 0.004, respectively).

**Table 4:** Distinct regulatory profiles of RNA by group throughout the study period.

Gene id	Function	Sedentary				Handball Athletes				*η*_*p*_^*2*^	P value Time	*η*_*p*_^*2*^	P value Time*Group
		T8 vs T0		T16 vs T8		T8 vs T0		T16 vs T8					
		Fold	Sd	Fold	Sd	Fold	Sd	Fold	Sd				
IRAK1	Acute inflammation	1.01	0.64	1.38	0.57	1.15	0.29	-1.68	0.44	0.01	0.53	0.10	0.02
CD81	Cell communication	-1.11	0.74	1.55	0.53	1.06	0.39	-1.59	0.52	0.01	0.38	0.01	0.02
ITGB1	Cell communication	-1.06	0.79	1.36	0.62	-1.08	0.46	-1.59	0.46	0.10	0.00	0.13	0.01
ACADS	Cell energy metabolism	-1.34	0.61	1.38	0.53	1.58	0.33	-1.89	0.36	0.00	0.63	0.13	0.01
PDHA2	Cell energy metabolism	-1.12	0.08	-1.25	0.11	2.35	0.08	-2.20	0.07	0.13	0.00	0.02	0.58
ACVR1B	Muscle growth/size/body function	-1.13	0.34	1.49	0.42	-1.09	0.47	-1.58	0.47	0.11	0.00	0.07	0.08
GPX1	Oxidative stress	1.01	0.62	1.76	0.44	1.03	0.40	-1.20	0.56	0.01	0.44	0.12	0.00
MTHFR	Vitamin metabolism/transport	1.06	0.71	1.18	0.63	1.24	0.41	-1.54	0.44	0.02	0.28	0.09	0.03
THTPA	Vitamin metabolism/transport	1.06	0.54	1.24	0.56	1.56	0.42	-1.50	0.46	0.03	0.14	0.07	0.04

FC, fold change, calculated as the ratio of RNA levels in athletes and sedentary controls at T0 (baseline conditions), T8 (after 8-weeks of multivitamin/mineral supplementation) and T16 (after 8-weeks in the absence of nutritional intervention). Data are for mean and standard deviation fold changes. The cut-off score to establish a significant fold change was established as 1.5. Partial eta squared (*η*^*2*^) effect size calculations were also performed to determine the effect of nutritional intervention and the interaction between the time*group (effect size: small, ≤0.01; moderate, 0.06; large, ≤0.14) [35]. Differences were considered significant at *p*-values < 0.05.

Time x group interaction existed for the sports performance genes IRAK1, CD81, ITGB1, ACADS, PDHA2 and GPX1 (ηP2 = 0.070 to 0.092; p < 0.05), as well as for the nutritional genes MTFR and THTPA (ηP2 = 0.070 to 0.092; p < 0.05).

**Fig 4:**
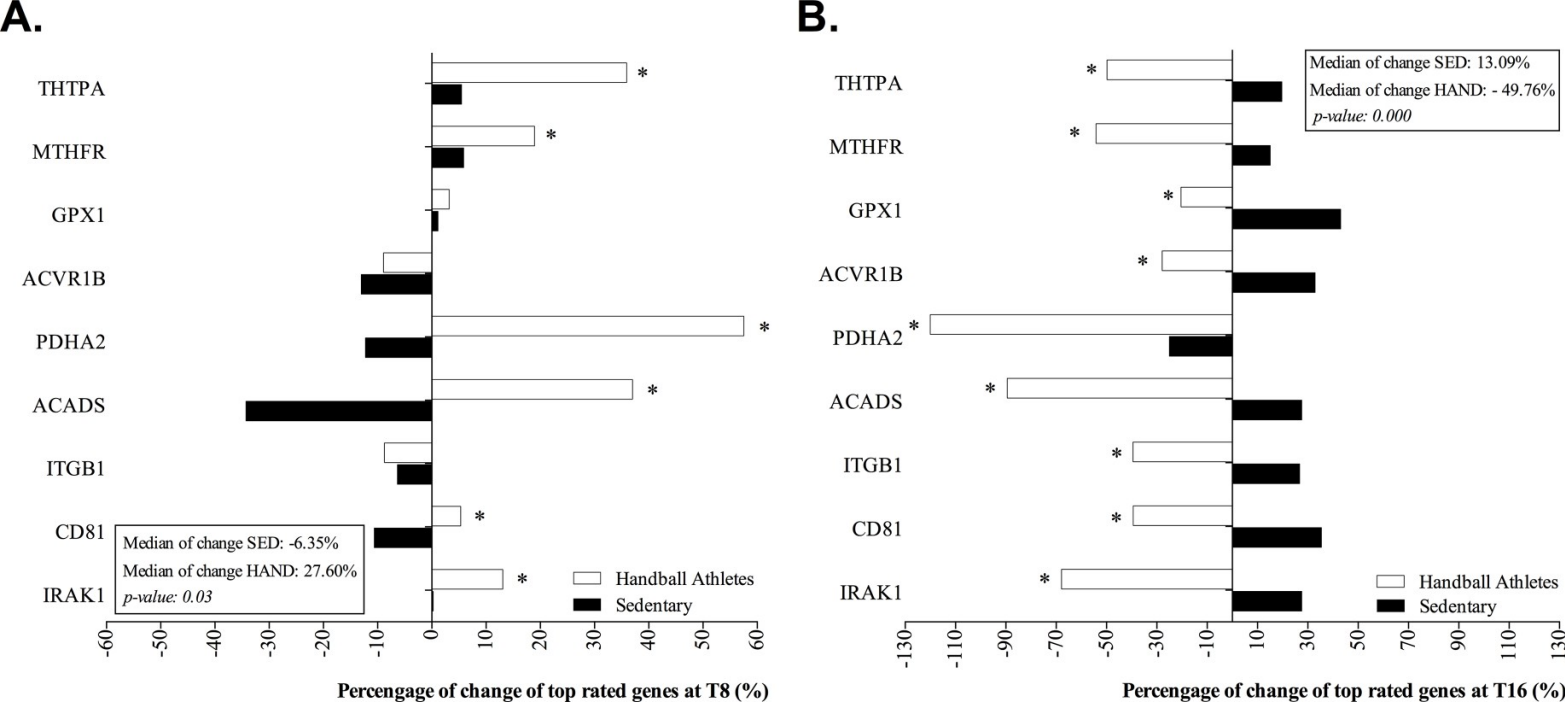
(A) Percentage change in gene expression for top-rated genes in handball athletes and sedentary controls from baseline to after 8 weeks of nutritional intervention (T8) and (B) after 8 weeks in the absence of nutritional intervention (T16).

**Fig 4** shows the percentage changes in RNA expression in top-rated genes of the handball athletes compared to the sedentary controls relative to baseline, analyzed at both T8 and T16. In the handball athletes, the 6 genes that were significantly affected by the intervention were upregulated from baseline values (27.60% [max: 43.1%, min: -24.9%]) and versus the sedentary controls (median gene expression in top-rated genes: -6.35% [max: 5.88%, min: -34.2%]). In contrast, downregulation was observed for 9 genes in the handball athletes compared to the sedentary controls after 8 weeks in the absence of nutritional intervention (median gene expression in top-rated genes: -56.7% [max: -20.41%, min: -120.0%] versus 13.09% [max: 57.5%, min: -8.91%]).

## Discussion

The present study has examined the modulation of gene expression in athletes under baseline conditions, and has analyzed the effect of a multivitamin/mineral supplementation intervention upon gene response in both elite handball athletes and in age- and gender-matched sedentary controls. Handball is considered a demanding exercise that generates important aerobic and anaerobic metabolic stress. Handball involves a large number of repetitive, intense eccentric muscle actions such as body contact, repeated decelerations, sprints, jumps, throwing, blocking, pushing and rapid changes in moving directions [36] that are associated to exercise induced muscle damage [37]. The major finding of this study is that a total of 7 key genes involved in cell communication, muscle function, oxidative stress response and immune function and inflammation were expressed differently in elite athletes compared to sedentary controls. Multivitamin/mineral supplementation modified the expression of the IRAK1, CD81, ITGB1, ACADS, PDHA2, ACRVB1, GPX1, MTHRF and THTPA genes differently in handball athletes and sedentary controls. Thus, our results partially confirm our hypotheses, primarily due to the initial differences between the groups that were observed in those genes related to sports performance—though a lesser impact was observed in those genes involved in metabolism and nutrient transport after the intervention.

Professional sports training involves repeated bouts of exercise and a large volume of physically demanding practice sessions and competitive games, which may lead to a decline in performance, with oxidative stress and inflammation [37]. Keeping in mind that blood connects the entire biological system, the expression profiles of circulating blood cells contain a specific signature in response to various physiological, pathological and environmental changes [10,38]. Our data suggest that ITGB1, ACVR1B, IL6R, SOD2 and MAPKAPK2 were found to be upregulated in handball players at baseline. In agreement with our findings, MAPKAPK2 has previously been shown to be significantly upregulated in peripheral blood in athletes after endurance exercise [12]. It seems to be involved in several cellular processes, including an increase in IL-6 synthesis through inflammation [39], which could explain the synergistic response of both genes observed in our study. Similarly, the reactive oxygen species-induced adaptive response following regular long-term training leads to upregulation of the antioxidant enzymatic systems, including superoxide dismutase. In this regard, Koltai et al. [40] showed antioxidant capacity through SOD2 in the skeletal muscle of elite athletes compared to control subjects. Thus, the upregulation of SOD2 in athletes in our study is consistent with the findings of previous studies.

There is a cellular rationale to suggest that the ACVR1B gene is linked to strength [41] and sprint/power [42] in athletes. Strength and power capabilities play a key role in team sports performance, and based on the scientific literature, specific physical training includes strength and power training that significantly influences jumping and throwing performance as well as physical confrontations in team handball [43]. ACVR1B is part of the TGF-β superfamily, a set of growth factors that regulate the expression level of certain genes implicated in muscle growth [44]. Although the existing literature has focused on study of the ACVR1B gene linkage to determine variations in muscle strength phenotypes in athletes [41], our study enhances the understanding of a greater expression profile of this gene in elite athletes as a response to exercise demands. At the cellular communication and inflammation level, the ITGB1 gene showed a percentage change between 53.9–65.2%. In accordance with our study, Lui et al. [11] found that exercise training can chronically induce transcriptional changes in peripheral blood. Those genes may be crucial in the alteration of the immunological state in highly trained individuals.

On the other hand, the cell energy metabolism and immune/inflammation genes, PDHA2 and MAPK11 (P38), were downregulated in handball athletes under baseline conditions. In contrast to our results, Desplanches et al. [45] reported the overexpression of PDHA2 in skeletal muscle in voluntary subjects shortly after exercise under conditions of normoxia. Although there are no studies reporting a decrease in PDHA2 gene expression, Hoffman et al. [46] recently revealed an extensive breadth of kinases regulated by exercise, where the PDHK family and PDHA2 as a member of PDHK were downregulated in human skeletal muscle. In relation to p38-MAPK, these are typically referred to as stress-activated protein kinases, since these MAPK are activated by mechanical tension, proinflammatory cytokines and oxidative stress [47]. In this regard, Nicoll et al. [48] suggested that MAPK activity may be altered during catabolic states such as overtraining, which may partially explain muscle maladjustment associated to stressful training. Thus, the decrease in gene expression of the p38 MAPK 11 gene could be an effect of the adaptive response to the competitive phase of the season in which our handball athletes were involved. It has been shown that the competition phase may lead to losses in lean body mass during the season, which is unfavorable for most power sports such as handball [49].

Our second aim was to explore possible modulation in gene expression after a nutritional intervention combining multivitamins and minerals. Our athletes showed maximum gene regulation effects after 8 weeks of supplementation (**Fig 2**). The two main genes affected by time changes were PDHA2 and ACADS—both being involved in cell energy metabolism. The pyruvate dehydrogenase complex catalyzes the overall conversion of pyruvate to acetyl coenzyme A and CO2, and thereby links the glycolytic pathway to the tricarboxylic cycle. To date, some authors [50] have suggested that exercise-trained subjects present an increased maximal capacity to use carbohydrates as a result of an increased expression of pyruvate dehydrogenase complex in skeletal muscle. Similarly, the ACADS gene is essential for fatty acid oxidation—the multistep process that breaks down fats and converts them into energy. In this regard, Zoladz et al. [51] have reported increased levels of ACADS (by ~approximately 65%) in isolated mitochondria from trained rats. This suggests that endurance training leads to upregulation of muscle mitochondrial oxidative capacity under physiological conditions. However, it is worth highlighting that in the present study, the ACADS gene seemed to be overexpressed by about 37% in athletes compared to baseline, and downregulated by about 90% after cessation of the nutritional intervention (timepoint T16 versus timepoint T8). This might explain the increased gene expression, and could reinforce the benefits resulting from adaptive changes involved in specific metabolic and physiologic processes in athletes [7], and the positive effect of the supplementation, as confirmed by the statistical significance of the observed time*group interaction (**Table 4**).

The IRAK1, CD81, ITGB1, MTHFR and THTPA genes, which are mainly involved in cell communication, acute inflammation and vitamin metabolism/transport, were counter-regulated 8 weeks after the athletes had stopped the nutritional treatment (**Fig 4B**). IRAK1 encodes for interleukin-1 receptor-associated kinase 1, one of two putative serine/threonine kinases that become associated with IL1R upon stimulation. Neubauer et al. [52] pondered the mechanisms by which neutrophils appear to counter-regulate proinflammatory signaling in response to intense exercise. Within this signaling cascade, IRAK3 would inhibit IL-1R and TLR signaling, possibly by interrupting the downstream signaling mediated by the interaction between IRAK1 and IRAK4 by finally inducing NF-kappaß and MAPK pathway activation. Therefore, due to the time*group interaction, we suggest that supplementation may restrict the response of the IRAK1 gene to reduce inflammation in athletes. In reference to the CD81 gene, Zieker et al. [12] suggested a possible important role of CD81 and other integrins in reacting or adapting to strenuous exercise in half-marathon runners. Specifically, CD81 downregulation may result in a lowered defense against infectious agents immediately following exercise [53], as a result of the alteration in the number and function of circulating innate immune system cells [4]. In endurance athletes, a low regulation of certain post-exercise integrins supported the hypothesis of an "open window" during the immediate post-exercise period in which a reduced ability to fight infectious agents was shown [53]. Another study referred to endurance activities such as the duathlon has confirmed the efficacy of a nutritional intervention with vitamin C, which would influence the expression response of some integrins such as MAC-1 to exercise-induced oxidative stress, increasing the activation of neutrophils [54]. Therefore, our results could support the idea that supplementation could have favored improved defense against infectious agents.

Researchers have long recognized the interaction between responses induced by exercise and nutrient availability. Indeed, training response adaptation by altering substrate supply has been a key research area for several decades [27]. Contrary to our expected results, we only found MTHFR and THTPA to modulate their expression at the end of the nutritional intervention and after 8 weeks in the absence of intervention, respectively. THTPA encodes for an enzyme which catalyzes the biosynthesis of thiamine diphosphate through the hydrolysis of thiamine triphosphate. In cultured human lymphocytes and fibroblasts, thiamine and its derivatives showed downregulated expression of the genes that encode for these proteins under deficient conditions [55]. In light of this, the effect of a short-term nutritional intervention was revealed to modify the expression of THTPA among other genes both in overweight and in lean subjects [56]. The results would imply that the THTPA genes may play an important role in the regulation of metabolic responses after nutritional intervention, though the regulatory mechanisms in response to nutritional status remain an open question [57]. Regarding MTHFR, Dinc et al. [58] analyzed genetic variation as a genetic marker of Hcy metabolism. However, to date no studies have explored MTHFR gene expression changes in athletes. No other vitamin- and mineral-related genes showed significant changes throughout the study intervention.

This study presents a number of results that fit very well with our current understanding of whole blood gene expression regulation through exercise and nutritional intervention using a highly sensitive, specific and accurate technique that allows the analysis of gene expression, comparing steady-state RNA levels between athletes and sedentary controls with previous internal standard selection. The use of well-evaluated reference genes in gene expression experiments reinforces the present results, which is particularly relevant when using high-throughput platforms like the QuantStudio 12K Flex Real-Time PCR System. In order to optimize our results, the sample was composed of subjects from the same handball team, who therefore had a homogeneous training intervention. Nonetheless, a limitation of our study is the limited number of participants, as is the case in many OpenArray studies. The high cost of this technology often precludes the inclusion of larger cohorts. In addition, the candidate genes involved in nutrient transport and metabolism showed a limited gene response, so the administration of a multivitamin/mineral supplement made it difficult to select relevant genes in the transport and metabolism of each micronutrient from the supplement. In this regard, and given that the literature supports the evidence of increased nutritional needs in athletes [59,60], we aimed to determine possible initial differences not only concerning the genes involved in sports performance, but also at nutritional level. Although there are interventional studies with multivitamin/mineral supplements in sports that show beneficial effects of supplementation, the approach from a molecular perspective should include a greater number of genes involved in the specific metabolic pathways of each nutrient, or alternatively isolated supplementation should be administered to enhance understanding of specific gene regulation in the transport and metabolism of a given nutrient.

## Conclusions

In sum, we have recorded a different expression profile of key genes in blood cells which are involved in major sports performance functions in elite handball players compared to sedentary controls. The multivitamin/mineral intervention showed a limited effect upon a few nutritional metabolic genes. However, the intervention modulated the coordinated up- or downregulation of key genes related to specific pathways and could have significant positive influences upon sports performance functions such as mitochondrial function, cell communication, muscle growth, oxidative stress response and acute inflammation.

## Supporting information

S1 Table Proportions of the vitamin and mineral complex supplement and their respective percentage contribution to the Recommended Daily Allowance (% RDA) estimated for adults. (DOCX)

S2 Table List of 112 TaqMan assays used for RT-qPCR analysis with the QuantStudioTM 12K Flex Real-Time PCR System. (DOCX)

S3 Table Stability values of 52 candidate reference genes for human total blood samples ranked by geNorm. (DOCX)

S1 Fig Flowchart of participants recruited, enrolled and involved in the study. (DOC)
