# Supplementary material for: Effect of 8-week of dietary micronutrient supplementation on gene expression in elite handball athletes
Source: PLoS One. 2020 May 1;15(5):e0232237. doi: 10.1371/journal.pone.0232237 (PMC7194438; doi:10.1371/journal.pone.0232237)
Supplement: S1 Table — (DOCX) [file pone.0232237.s001.docx]

**S1 Table.** Proportions of the vitamin and mineral complex supplement and their respective percentage contribution to the Recommended Daily Allowance (% RDA) estimated for adults.

| **Ingredients** | **Units** | **Amount** | **% of RDA** | **EU RDA** |
| --- | --- | --- | --- | --- |
| Vitamin A | μg | 800 | 107 | 750 |
| Vitamin E | mg | 18 | 138 | 13 |
| Vitamin C | mg | 120 | 109 | 110 |
| Vitamin K | μg | 30 | 43 | 70 |
| Vitamin B1 | mg | 1.6 | 145 | 1.1 |
| Vitamin B2 | mg | 2.1 | 162 | 1.3 |
| Vitamin B6 | mg | 2.1 | 124 | 1.7 |
| Vitamin B12 | μg | 3 | 75 | 4 |
| Vitamin D | μg | 5 | 100 | 5 |
| Biotin | μg | 62.5 | 156 | 40 |
| Folate | μg | 200 | 80 | 250 |
| Niacin | mg | 20 | 125 | 16 |
| Pantothenic acid | mg | 7.5 | 150 | 5 |
| Calcium | mg | 200 | 21 | 950 |
| Phosphorous | mg | 105 | 19 | 550 |
| Magnesium | mg | 120 | 40 | 300 |
| Iron | mg | 3.75 | 23 | 16 |
| Iodine | μg | 100 | 67 | 150 |
| Cooper | μg | 500 | 38 | 1,300 |
| Manganese | mg | 2 | 67 | 3 |
| Chrome | μg | 40 | 100 | 40 |
| Molybdenum | μg | 50 | 77 | 65 |
| Selenium | μg | 30 | 43 | 70 |
| Zinc | mg | 5 | 39 | 12.7 |

* European recommended daily allowance in accordance to European Food Safety Authority (EFSA) (59).
