# Supplementary material for: Effect of 8-week of dietary micronutrient supplementation on gene expression in elite handball athletes
Source: PLoS One. 2020 May 1;15(5):e0232237. doi: 10.1371/journal.pone.0232237 (PMC7194438; doi:10.1371/journal.pone.0232237)
Supplement: S2 Table — (DOCX) [file pone.0232237.s002.docx]

**S2 Table. List of 112 TaqMan assays used for RT-qPCR analysis with the QuantStudioTM 12K Flex Real-Time PCR System.**

| **Assay id** | **Gene Symbol** | **Gene Name** |
| --- | --- | --- |
| Hs00163506_m1 | ACADS | Acyl-CoA dehydrogenase, C-2 to C-3 short chain |
| Hs00174179_m1 | ACE | Angiotensin I converting enzyme (peptidyl-dipeptidase A) 1 |
| Hs00960561_m1 | ACSL1 | Acyl-CoA synthetase long-chain family member 1 |
| Hs00153812_m1 | ACTN3 | Actinin, alpha 3 |
| Hs00244715_m1 | ACVR1B | Activin A receptor, type IB |
| Hs00176119_m1 | AK1 | Adenylate kinase 1 |
| Hs00193059_m1 | ALDOC | Aldolase C, fructose-bisphosphate |
| Hs00734523_m1 | ARF1 | ADP-ribosylation factor 1 |
| Hs00969569_m1 | ATP5B | ATP synthase H+ transporting, mitochondiral F1 complex, beta polypeptide |
| Hs00363176_m1 | BCMO1 | Beta-carotene 15,15'-monooxygenase 1 |
| Hs00537320_m1 | BTNL9 | Butyrophilin-like 9 |
| Hs00156308_m1 | CAT | Catalase |
| Hs00174360_m1 | CD130 (pf130) | Il-6 signal transducer |
| Hs00990732_m1 | CD34 | CD34 molecule |
| Hs01567185_m1 | CD36/FAT | CD36 molecule (thrombospondin receptor) |
| Hs00174717_m1 | CD81 | CD81 molecule |
| Hs00426680_mH | CDC20 | Cell division cycle 20 homolog |
| Hs00176490_m1 | CKM | Creatine kinase, muscle |
| Hs00266237_m1 | COL4A1 | Collagen, type IV, alpha 1 |
| Hs00542284_m1 | CTH | Cystathionase (cystathionine gamma-lyase) |
| Hs00426608_m1 | CYP4F2 | Cytochrome P450, family 4, subfamily F, polypeptide 2 |
| Hs00758822_s1 | DHFR | Dihydrofolate reductase |
| Hs01124177_m1 | FOLR1 | Folate receptor 1 |
| Hs01694011_s1 | FTH1 | Ferritin, heavy polypeptide 1 |
| Hs00609799_m1 | GCKR | Glucokinase (hexokinase 4) regulator |
| Hs00914163_m1 | GGH | Gamma-glutamyl hydrolase (conjugase, folylpolygammaglutamyl hydrolase) |
| Hs00792200_g1 | GH1 | Growth hormone 1 |
| Hs00168966_m1 | GLUT4/SLC2A4 | Solute carrier family 2(facilitated glucose transporter), member4 |
| Hs00829989_gH | GPX1 | Glutathion peroxidase I |
| Hs00989766_g1 | GPX4 | Glutathione peroxidase 4 |
| Hs00167317_m1 | GSR | Glutathione reductase |
| Hs00157863_m1 | GYS1 | Glycogen synthase 1 (muscle) |
| Hs00262142_g1 | H19 | H19, imprinted maternally expressed transcript |
| Hs00373474_m1 | HFE | Hemochromatosis |
| Hs00153153_m1 | HIF1A | Hypoxia inducible factor 1, alpha subunit |
| Hs00941880_m1 | Hsc70 | Heat Shock protein 70kDa protein 8 |
| Hs03044127_g1 | HSPB1/HSP27 | Heat shock 27kDa protein 1 |
| Hs00989291_m1 | IFNG | Interferon; gamma |
| Hs00988304_m1 | IFNGR1 | Interferon gamma receptor 1 |
| Hs01547656_m1 | IGF1 | Insulin-like growth factor 1 (somatomedin C) |
| Hs04188276_m1 | IGF2 | Insulin-like growth factor 2 |
| Hs04188276_m1 | IGF2 | Insulin-like growth factor 2 |
| Hs00376160_m1 | IGJ | Immunoglobulin J polypeptide, linker protein for immunoglobulin alpha |
| Hs00961622_m1 | IL-10 | Interleukin 10 |
| Hs00174114_m1 | IL-2 | Interleukin 2 |
| Hs00174122_m1 | IL-4 | Interleukin 4 |
| Hs01555410_m1 | IL1B | Interleukin 1 |
| Hs00893626_m1 | IL1RN | Interleukin 1 receptor antagonist |
| Hs00985639_m1 | IL6 | Interleukin 6 |
| Hs01075666_m1 | IL6R (cd126) | IL-6 receptor |
| Hs01018347_m1 | IRAK1 | Interleukin-1 receptor-associated kinase 1 |
| Hs00174217_m1 | ITGAX | Integrin alpha X (complement component 3 receptor 4 subunit) |
| Hs00164957_m1 | ITGB1 | Integrin beta 2 |
| Hs01026983_m1 | JACK1 | Janus kinase 1 |
| Hs00234567_m1 | JACK2 | Janus kinase 2 |
| Hs00358836_m1 | KLF4 | Kruppel-like factor 4 (gut) |
| Hs00193510_m1 | LIPE | Lipase, hormone-sensitive |
| Hs00173425_m1 | LPL | Lipoprotein lipase |
| Hs00255388_m1 | LUC7L2 | LUC7L2-like 2 |
| Hs01558722_m1 | MAPK11 (P38) | Mitogen-activated protein kinase-activated protein kinase 11 |
| Hs01116946_m1 | MAPKAP K2 | Mitogen-activated protein kinase-activated protein kinase 2 |
| Hs00538861_m1 | MT1B | Metallothionein 1B |
| Hs00744661_sH | MT1F | Metallothionein 1F |
| Hs02578922_gH | MT1G | Metallothionein 1G |
| Hs00823168_g1 | MT1H | Metallothionein 1H |
| Hs02379661_g1 | MT2A | Metallothionein 2A |
| Hs01921768_s1 | MT3 | Metallothionein 3 |
| Hs01068263_m1 | MTHFD1 | Methylenetetrahydrofolate dehydrogenase (NADP+ dependent) 1 |
| Hs00914916_m1 | MTHFD1L | Methylenetetrahydrofolate dehydrogenase (NADP+ dependent) 1-like |
| Hs00195560_m1 | MTHFR | Methylenetetrahydrofolate reductase (NAD(P)H) |
| Hs00165188_m1 | MTR | 5-methyltetrahydrofolate-homocysteine methyltransferase |
| Hs00985015_m1 | MTRR | 5-methyltetrahydrofolate-homocysteine methyltransferase reductase |
| Hs00267293_m1 | MYH8 | Myosin, heavy chain 8, skeletal muscle, perinatal |
| Hs00414907_m1 | MZB1 | Marginal zone B and B1 cell-specific protein |
| Hs00413685_m1 | PAPOLA | Poly(A) polymerase alpha |
| Hs01043024_s1 | PDHA2 | Pyruvate dehydrogenase (lipoamide) alpha 2 |
| Hs01037712_m1 | PDK4 | Pyruvate dehydrogenase kinase, isozyme 4 |
| Hs00947536_m1 | PPARA | Peroxisome proliferator-activated receptor alpha |
| Hs01016719_m1 | PPARGC1A | Peroxisome proliferator-activated receptor gamma, coactivator 1 alpha |
| Hs00991677_m1 | PPARGC1B | Peroxisome proliferator-activated receptor gamma, coactivator 1 beta |
| Hs00738905_g1 | PRDX5 | Peroxiredoxin 5 |
| Hs01562315_m1 | PRKAA1 | Protein kinase, AMP-activated, alpha 1 catalytic subunit |
| Hs00177357_m1 | RPS6KB1 | Ribosomal protein S6 kinase |
| Hs00225345_m1 | SBP2 | SECIS binding protein 2 |
| Hs00969821_m1 | SCARB1 | Scavenger receptor class B, member 1 |
| Hs00174151_m1 | SELL | Selectin L |
| Hs01032845_m1 | SEPP1 | Selenoprotein P, plasma, 1 |
| Hs00541038_m1 | SHMT1 | Serine hydroxymethyltransferase 1 (soluble) |
| Hs00167206_m1 | SLC11A2 | Solute carrier family 11 (proton-coupled divalent metal ion transporters), member 2 |
| Hs00953344_m1 | SLC19A1 | Solute carrier family 19 (folate transporter), member 1 |
| Hs00949696_m1 | SLC19A2 | Solute carrier family 19 (thiamine transporter), member 2 |
| Hs00228858_m1 | SLC19A3 | Solute carrier family 19, member 3 |
| Hs00253602_m1 | SLC30A1 | Solute carrier family 30 (zinc transporter), member 1 |
| Hs00545183_m1 | SLC30A8 | Solute carrier family 30 (zinc transporter), member 8 |
| Hs00606016_g1 | SLC52A1 | Solute carrier family 52, riboflavin transporter, member 1 |
| Hs00364295_m1 | SLC52A3 | Solute carrier family 52, riboflavin transporter, member 3 |
| Hs02330328_s1 | SOCS3 | Suppressor of cytokine signaling 3 |
| Hs00533490_m1 | SOD1 | Superoxide dismutase 1 |
| Hs00167309_m1 | SOD2 | Superoxide dismutase 2 |
| Hs01013996_m1 | STAT1 | Signal transducer and activator of transcription 1, 91 kDa. |
| Hs00374280_m1 | STAT3 | Signal transducer and activator of transcription 3. |
| Hs00370305_m1 | TANK | TRAF family member-associated NFKB activator |
| Hs01055542_m1 | TCN1 | Transcobalamin I (vitamin B-12 binding protein, R binder family) |
| Hs00165902_m1 | TCN2 | Transcobalamin II |
| Hs00225677_m1 | THTPA | Thiamine triphosphatase |
| Hs01558699_m1 | TPK1 | Thiamin pyrophosphokinase 1 |
| Hs01019353_m1 | TRPM6 | Transient receptor potential cation channel, subfamily M, member 6 |
| Hs00918956_m1 | TRPM7 | Transient receptor potential cation channel, subfamily M, member 7 |
| Hs00609398_m1 | TTPA | Alpha-tocopherol transfer protein |
| Hs01046710_m1 | TXNDC5 | Thioredoxin domain containing 5 (endoplasmic reticulum) |
| Hs01106052_m1 | UCP3 | Uncoupling protein 3 (mitochondrial, proton carrier) |
| Hs00172113_m1 | VDR | Vitamin D (1,25- dihydroxyvitamin D3) receptor |
