# Supplementary material for: Effect of 8-week of dietary micronutrient supplementation on gene expression in elite handball athletes
Source: PLoS One. 2020 May 1;15(5):e0232237. doi: 10.1371/journal.pone.0232237 (PMC7194438; doi:10.1371/journal.pone.0232237)
Supplement: S3 Table — (DOCX) [file pone.0232237.s003.docx]

**Supplementary Table 3.** Stability values of 52 candidate reference genes for human total blood samples ranked by geNorm.

| **Gene symbol** | **Assay id** | **Stability value (M)** | **Coefficient of variation (CV)** |
| --- | --- | --- | --- |
| ARF1 | Hs00734523_m1 | 0.177 | 0.013 |
| PAPOLA | Hs00413685_m1 | 0.177 | 0.008 |
| ATP5B | Hs00969569_m1 | 0.181 | 0.023 |
| HSP90AB1 | Hs01546474_g1 | 0.181 | 0.036 |
| POLR2A | Hs00172187_m1 | 0.181 | 0.028 |
| RPL37A | Hs01102345_m1 | 0.181 | 0.031 |
| IPO8 | Hs00183533_m1 | 0.182 | 0.042 |
| CDKN1B | Hs00153277_m1 | 0.184 | 0.037 |
| RPLP0 | Hs00420895_gH | 0.188 | 0.036 |
| TRIM27 | Hs00179059_m1 | 0.189 | 0.046 |
| FBXW2 | Hs01004164_m1 | 0.195 | 0.058 |
| G6PD | Hs00166169_m1 | 0.196 | 0.047 |
| POP4 | Hs00198357_m1 | 0.196 | 0.062 |
| ACTB | Hs99999903_m1 | 0.201 | 0.067 |
| ELF1 | Hs00152844_m1 | 0.201 | 0.078 |
| MRPL19 | Hs00608519_m1 | 0.201 | 0.079 |
| UBC | Hs00824723_m1 | 0.203 | 0.056 |
| EIF2B1 | Hs00426752_m1 | 0.206 | 0.086 |
| PPIA | Hs03045347_gH | 0.208 | 0.067 |
| LUC7L2 | Hs00255388_m1 | 0.209 | 0.090 |
| SDHA | Hs00188166_m1 | 0.209 | 0.089 |
| MYL6 | Hs00819642_m1 | 0.213 | 0.071 |
| UBE2D2 | Hs00366152_m1 | 0.213 | 0.084 |
| HPRT1 | Hs01003267_m1 | 0.215 | 0.087 |
| GUSB | Hs99999908_m1 | 0.221 | 0.081 |
| RPL30 | Hs00265497_m1 | 0.228 | 0.110 |
| PPIA | Hs99999904_m1 | 0.229 | 0.100 |
| RPLP0 | Hs99999902_m1 | 0.229 | 0.090 |
| PUM1 | Hs00206469_m1 | 0.231 | 0.112 |
| ALAS1 | Hs00167441_m1 | 0.232 | 0.092 |
| CALM2 | Hs00830212_s1 | 0.233 | 0.095 |
| TBP | Hs99999910_m1 | 0.243 | 0.127 |
| DIMT1L | Hs00205515_m1 | 0.248 | 0.132 |
| MT-ATP6 | Hs02596862_g1 | 0.249 | 0.129 |
| GAPDH | Hs99999905_m1 | 0.251 | 0.116 |
| CASC3 | Hs00201226_m1 | 0.253 | 0.115 |
| YWHAZ | Hs00237047_m1 | 0.266 | 0.142 |
| PES1 | Hs00362795_g1 | 0.267 | 0.144 |
| HPRT1 | Hs99999909_m1 | 0.270 | 0.142 |
| ABL1;BCR | Hs00245445_m1 | 0.272 | 0.137 |
| PGK1 | Hs99999906_m1 | 0.288 | 0.152 |
| PSMC4 | Hs00197826_m1 | 0.302 | 0.166 |
| TFRC | Hs99999911_m1 | 0.302 | 0.185 |
| TFRC | Hs00174609_m1 | 0.315 | 0.198 |
| 18S | Hs03003631_g1 | 0.327 | 0.207 |
| CUL1 | Hs01117001_m1 | 0.328 | 0.192 |
| B2M | Hs99999907_m1 | 0.332 | 0.177 |
| OAZ1 | Hs00427923_m1 | 0.383 | 0.224 |
| 18S | Hs99999901_s1 | 0.394 | 0.260 |
| CDKN1A | Hs00355782_m1 | 0.441 | 0.277 |
| HMBS | Hs00609297_m1 | 0.476 | 0.320 |
| GAPDH | Hs00266705_g1 | 0.660 | 0.476 |
| GADD45A | Hs00169255_m1 | 0.697 | 0.494 |
