# Supplementary material for: Effect of 8-week of dietary micronutrient supplementation on gene expression in elite handball athletes
Source: PLoS One. 2020 May 1;15(5):e0232237. doi: 10.1371/journal.pone.0232237 (PMC7194438; doi:10.1371/journal.pone.0232237)
Supplement: S1 Fig — (DOC) [file pone.0232237.s004.doc]

**Supplemental Figure 1.** Flowchart of participants recruited, enrolled and involved in the study.

**Allocation**

**Analysis**

**Follow-Up**

**Enrollment**

Assessed for eligibility (n=32)

Allocated to intervention (n=14)

- Received allocated intervention (n=14)

Allocated to intervention (n=16)

- Received allocated intervention (n=13)

Excluded (n= 2)

- Not meeting inclusion criteria (n= 2)

Data analysed (n= 13)

- Completed full intervention (n= 13)

Lost to follow-up (give reasons) (n=1)

- Discontinued intervention (withdrew from the study due to injury) (n=1)

Lost to follow-up (give reasons) (n=3)

- Discontinued intervention (Supplementation commitment (n=2)
- Discontinued intervention (Time commitment (n=1)

) (n= )

Data analysed (n= 13)

- Completed full intervention (n= 13)
